# A Novel Approach to Cardiac Magnetic Resonance Scar Characterization in Patients Affected by Cardiac Amyloidosis: A Pilot Study

**DOI:** 10.3390/medicina60040613

**Published:** 2024-04-08

**Authors:** Michele Alfieri, Federico Guerra, Carla Lofiego, Marco Fogante, Giuseppe Ciliberti, Fabio Vagnarelli, Alessandro Barbarossa, Samuele Principi, Giulia Stronati, Giovanni Volpato, Paolo Compagnucci, Yari Valeri, Paolo Tofoni, Leonardo Brugiatelli, Irene Capodaglio, Paolo Esposto Pirani, Giulio Argalia, Nicolò Schicchi, Loredana Messano, Maurizio Centanni, Andrea Giovagnoni, Gian Piero Perna, Antonio Dello Russo, Michela Casella

**Affiliations:** 1 Cardiology and Arrhythmology Clinic, University Hospital “Azienda Ospedaliero-Universitaria delle Marche”, Via Conca 71, 60126 Ancona, Italygiuseppe.ciliberti@ospedaliriuniti.marche.it (G.C.); paolocompagnucci1@gmail.com (P.C.); s1117769@pm.univpm.it (Y.V.); p.tofoni@pm.univpm.it (P.T.); s1102285@pm.univpm.it (L.B.);; 2 Department of Biomedical Sciences and Public Health, Marche Polytechnic University, 60121 Ancona, Italy; 3 Division of Cardiology, University Hospital “Azienda Ospedaliero-Universitaria delle Marche”, Via Conca 71, 60126 Ancona, Italy; 4 Cardiovascular Radiological Diagnostics, Department of Radiological Sciences, University Hospital “Azienda Ospedaliero-Universitaria delle Marche”, Via Conca 71, 60126 Ancona, Italy; marco.fogante@ospedaliriuniti.marche.it (M.F.);; 5 Department of Clinical, Special and Dental Sciences, Marche Polytechnic University, 60121 Ancona, Italy

**Keywords:** cardiac magnetic resonance, magnetic resonance imaging, cardiac amyloidosis, ventricular arrhythmias, ATTR, AL

## Abstract

*Background and Objectives*: Cardiac magnetic resonance (CMR) imaging has become an essential instrument in the study of cardiomyopathies; it has recently been integrated into the diagnostic workflow for cardiac amyloidosis (CA) with remarkable results. An additional emerging role is the stratification of the arrhythmogenic risk by scar analysis and the possibility of merging these data with electro-anatomical maps. This is made possible by using a software (ADAS 3D, Galgo Medical, Barcelona, Spain) able to provide 3D heart models by detecting fibrosis along the whole thickness of the myocardial walls. Little is known regarding the applications of this software in the wide spectrum of cardiomyopathies and the potential benefits have yet to be discovered. In this study, we tried to apply the ADAS 3D in the context of CA. *Materials and Methods*: This study was a retrospectively analysis of consecutive CMR imaging of patients affected by CA that were treated in our center (Marche University Hospital). Wherever possible, the data were processed with the ADAS 3D software and analyzed for a correlation between the morphometric parameters and follow-up events. The outcome was a composite of all-cause mortality, unplanned cardiovascular hospitalizations, sustained ventricular arrhythmias (VAs), permanent reduction in left ventricular ejection fraction, and pacemaker implantation. The secondary outcomes were the need for a pacemaker implantation and sustained VAs. *Results*: A total of 14 patients were deemed eligible for the software analysis: 8 patients with wild type transthyretin CA, 5 with light chain CA, and 1 with transthyretin hereditary CA. The vast majority of imaging features was not related to the composite outcome, but atrial wall thickening displayed a significant association with both the primary (*p* = 0.003) and the secondary outcome of pacemaker implantation (*p* = 0.003). The software was able to differentiate between core zones and border zones of scars, with the latter being the most extensively represented in all patients. Interestingly, in a huge percentage of CMR images, the software identified the highest degree of core zone fibrosis among the epicardial layers and, in those patients, we found a higher incidence of the primary outcome, without reaching statistical significance (*p* = 0.18). Channels were found in the scar zones in a substantial percentage of patients without a clear correlation with follow-up events. *Conclusions*: CMR imaging plays a pivotal role in cardiovascular diagnostics. Our analysis shows the feasibility and applicability of such instrument for all types of CA. We could not only differentiate between different layers of scars, but we were also able to identify the presence of fibrosis channels among the different scar zones. None of the data derived from the ADAS 3D software seemed to be related to cardiac events in the follow-up, but this might be imputable to the restricted number of patients enrolled in the study.

## 1. Introduction

### 1.1. Amyloid Cardiomyopathy

Cardiac amyloidosis (CA) is a restrictive cardiomyopathy resulting from the deposition of misfolded proteins in the extracellular space of myocardial cells. It confers stiffness to the myocardial wall, resulting in elevated filling pressures and quickly evolving into heart failure with preserved ejection fraction (HFpEF). Further cardiological findings are aortic stenosis and arrhythmic events. Despite many efforts made in the development of valid diagnostic and therapeutic tools, the prognosis remains poor for most patients. The diagnostic process includes bone scintigraphy, serum and urine immunofixation, as well as the free light chain assay.

### 1.2. Cardiac Magnetic Resonance Imaging

In cases of uncertainty and to assess myocardial involvement in the light chain (AL) form of amyloidosis, cardiac magnetic resonance (CMR) imaging represents a valuable weapon in our arsenal. With its exclusive features of T1 and T2 mapping, extracellular volume (ECV) quantification, and late gadolinium enhancement (LGE) assessment, CMR can distinguish among a wide range of cardiomyopathies while providing reliable prognostic factors in the natural course of CA [1,2,3,4,5,6,7]. Among these, T1 is related to both the intra- and extracellular spaces and its evaluation after the administration of contrast gives the value of ECV, which is the earliest marker of disease and provides indications regarding the amount of amyloid infiltration. On the other hand, T2 mapping increases along with myocardial edema and is a good indicator of the treatment response in patients with AL-CA [5]. Lastly, left ventricular LGE is one of the most widely accepted prognostic factors for a plethora of cardiomyopathies and CA is no exception. It represents an expansion of the extracellular space (often due to fibrosis or inflammation) and its prevalence can reach up to 80% of patients affected by CA with two main recognizable patterns: subendocardial and transmural. The former is mainly located in basal and mid-ventricular regions; it is mostly prevalent in the AL variant of CA and can be enhanced by dark-blood acquisitions. On the other hand, the transmural pattern is a better predictor of adverse outcomes [8,9,10,11]. A less described feature is atrial LGE; this finding represents another important marker of CA that is frequently associated with atrial dysfunction and carries a high specificity for the disease [12].

### 1.3. A Novel CMR Tool

**Figure 1:**
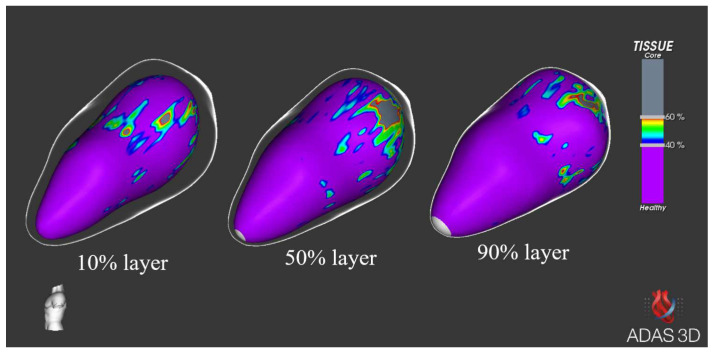
Images obtained and analyzed with the ADAS 3D software from the same patient. We are able to identify the tissue structure in every myocardial layer (10% endocardial layer, 50% intramyocardial layer, and 90% sub-epicardial layer).

**Figure 2:**
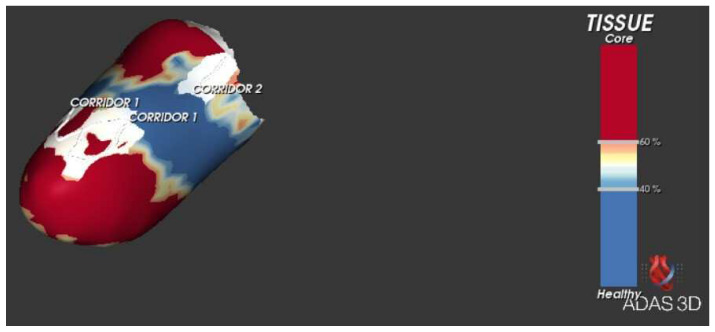
Image from the ADAS 3D software. Note the slow conduction channels (“corridors”) inside fibrotic zones.

One recent application of CMR is the ability of merging imaging data with electro-anatomical mapping systems. This is made possible by a software (ADAS 3D, Galgo Medical, Barcelona, Spain) that is capable of reconstructing the myocardial structure and identifying the presence, localization, and extension of scar zones in each myocardial layer (Figure 1). The software can address scar tissue and distinguish between core zones, made of dense scar, and border zones, which represent a mixture of healthy tissue and fibrosis, thus providing important information regarding electrophysiological structures. Furthermore, a specific module allows for the automatic detection of channels of healthy tissue inside scar areas that may serve as a substrate for arrhythmogenesis (Figure 2) [13]. This singular feature provides an accurate risk stratification without the need for an invasive evaluation and may therefore be crucial in the management of CA patients. In this study, we tried to prove the feasibility and potential application of such an instrument by correlating imaging findings with clinical events.

## 2. Materials and Methods

**Figure 3:**
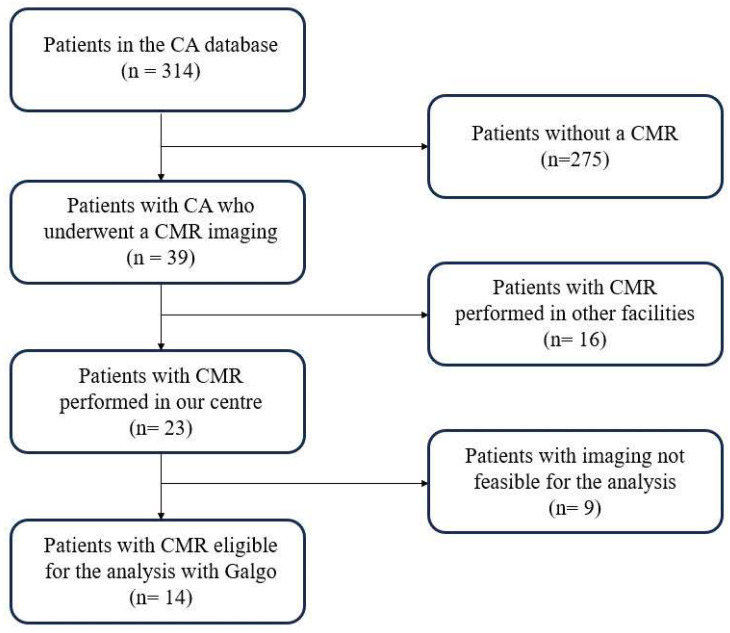
Selection process for the ADAS 3D software analysis.

This is a retrospective analysis of prospectively collected data from consecutive patients with a definite diagnosis of CA that were followed-up at our Center, Marche University Hospital, from March 2014 to November 2022. Figure 3 shows the study population enrolment flow-chart. The inclusion criteria were a definite diagnosis of CA, age > 18 years old, and having an available CMR with a gadolinium-based contrast. The exclusion criterion was the presence of poor imaging quality due to artifacts and movement. Patients whose CMR imaging date could not be found were excluded from the analysis. All patients underwent a full cardiological visit by expert cardiologists with an echocardiogram and ECG at baseline, while follow-up was conducted through a new visit, if deemed possible, or through telephone. This study was conducted according to institutional guidelines, national legal requirements, and the revised Declaration of Helsinki. The data collection was approved by the local ethics committee (n. 173/2022) and all patients provided written informed consent.

### 2.1. Cardiological Assessment

All patients underwent a complete cardiological evaluation including anamnesis, ECGs, and echocardiography at baseline. Every cardiological assessment was conducted by trained cardiologists and echocardiography was performed according to the appropriate guidelines. The treatment was adapted to every patient’s needs according to a clinical evaluation, available guidelines, and the best medical practice.

### 2.2. Endpoints

After the imaging analysis, we looked for a correlation between morphometric parameters and follow-up events. The date of the CMR imaging was taken as the follow-up starting point. The primary outcome was a composite of unplanned cardiovascular hospitalizations, all-cause mortality, sustained ventricular arrhythmias (VAs; defined as a ventricular tachycardia lasting more than 30 s, ventricular fibrillation, ventricular flutter, or sudden cardiac death), permanent reduction in left ventricular ejection fraction (EF), and the need for a pacemaker implantation. The secondary outcomes were the need for PMK implantation and sustained VAs.

### 2.3. Statistical Analysis

For all continuous variables, normality was tested through the Kolmogorov–Smirnov test. Variables with a normal distribution are represented as the mean and standard deviation. Non-normally distributed items are represented as the median and interquartile range (IQR). Categorical variables were described in terms of absolute and relative values.

To assess the incidence of the primary and secondary endpoints, a Kaplan–Meier analysis was employed. To analyze the association between baseline characteristics and the primary outcome, the variables that showed an association with each endpoint with a significance level < 0.1 according to univariate analyses were entered into the multivariable Cox proportional model. The statistical analyses were performed by using SPSS 25.0 for Windows (SPSS Inc., Chicago, IL, USA). A value of *p* < 0.05 (two-tailed) was considered statistically significant.

### 2.4. CMR Protocol

All the CMR exams followed a standardized protocol. Scouting images in the axial, coronal, and sagittal planes were utilized for planning subsequent cardiac views. Half-Fourier acquisition single-shot turbo spin echo (HASTE) images were taken through the chest with the parameters set at a TR/TE of 1500/30. Cine steady-state free precession (SSFP) images were obtained in the vertical long-axis, short-axis, and four-chamber views, with TR/TE set at 2.8/1.4, a flip angle of 70°, a slice thickness of 8 mm, a gap of 0 mm. T2-weighted double-inversion fast spin echo (FSE) images were acquired in the short-axis view, with parameters including a TR/TE of 2000/80, a TR of 2 or 3 R-R intervals, an inversion time (TI) of 150 ms, a section thickness of 8 mm, a gap of 0 mm, a matrix size of 256 × 256, and an echo-train length of 20 to 32. Inversion recovery delayed enhanced images were taken 8 min post-injection of 0.2 mmol/kg of gadolinium. Prior to this sequence, a “Look-Locker” sequence was performed to determine the optimal inversion time, with the parameters set at a TR/TE of 8/3, a flip angle of 12°, 1 slice repeated 32 times, and a slice thickness of 10 mm. A segmented phase-sensitive inversion recovery (PSIR) gradient-echo sequence was acquired in the short-axis and four-chamber views, with a TR/TE of 6.5/3.2, a trigger pulse of 2, a flip angle of 15°, an average number of slices ranging from 8 to 12, a slice thickness of 8 mm, and a gap of 2 mm.

### 2.5. Software Application

CMR imaging was performed when indicated according to the proper guidelines and clinical suspicion. Only CMR images performed with gadolinium-based contrast in our center were selected. In patients where CMR imaging was deemed feasible, the ADAS 3D software was employed. For an optimal ventricular analysis with ADAS 3D, the recommended CMR magnetic field is 1.5 or 3 T with sequences taken from 10 to 20 min after contrast injection. Through the application of such software, three-dimensional models of the ventricular walls are made by representing layers of the left ventricular thickness. Areas with a high enhancement are referred to as core zones, while low enhanced areas are defined as healthy tissue. A corridor was identified as a path of border zone tissue starting and ending in a healthy tissue area travelling between regions of core zones. The software recognizes valves and the epicardial surface as core zones and those areas are considered as “boundaries” for corridor detection.

## 3. Results

### 3.1. Population

**Figure 4:**
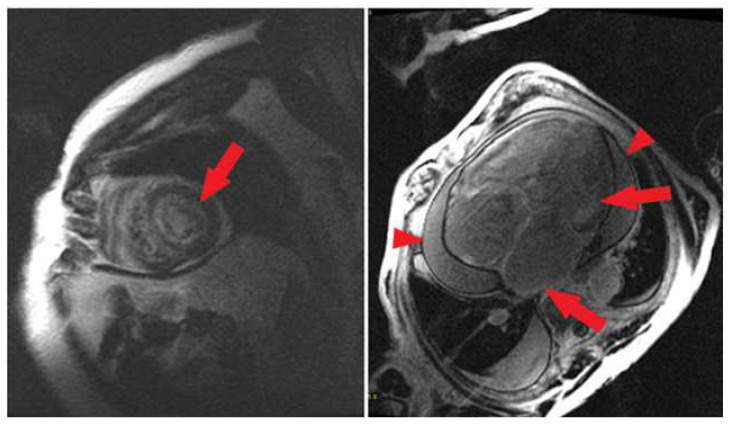
On the left, an example of subendocardial biventricular LGE (short axis). On the right, a case of subendocardial and bi-atrial LGE with pericardial effusion (4-chamber view). Red arrows represent LGEs, arrowheads indicate pericardial effusion.

For 39 patients (35 males, 89.7%; age at the time of CMR imaging: 69.9 ± 10.8 years old; 38 Caucasian patients, 97.4%), a CMR image with gadolinium contrast medium was available. A total of 32 patients were affected by wild type transthyretin (ATTR-wt) CA (82.1%), 6 patients by AL-CA (15.4%), and 1 patient (2.6%) by transthyretin hereditary (ATTR-m) CA. Out of all the exams, in 37 cases (94.9%), LGE was present, with subendocardial involvement as the most prevalent finding (*n* = 27; 69.2%; Figure 4), often present with further LGE patterns in the same patient (*n* = 7; 17.9%). Mid-wall and subepicardial patterns (*n* = 8; 20.5%) and the transmural pattern (*n* = 8; 20.5%) were less commonly represented. All patients with transmural LGE were affected by ATTR-wt.

Among patients who underwent CMR imaging, only the exams performed in our hospital were included (*n* = 23). In this cohort, we were able to detect a significant amount of atrial LGE in 21 patients (91.3%; Figure 4). After further evaluation, 9 CMR images were excluded from the analysis due to suboptimal imaging, thus leaving 14 patients eligible for the ADAS 3D software analysis (Figure 3).

The baseline characteristics of the final population are reported in Table 1. The mean follow-up time was 29.5 ± 22.2 months. Of the final 14 patients, 8 were affected by ATTR-wt (57.1%), 5 by AL (35.7%), and 1 by ATTR-m (7.1%). The vast majority was still represented by men (*n* = 11, 78.6%) and the mean age at the time of the CMR imaging was 67.6 ± 13.9 years. An LGE was detected in all CMR images: 1 patient (7.1%) had a transmural LGE pattern along with a subendocardial enhancement, 10 patients presented an isolated subendocardial pattern (71.4%), 1 patient (7.1%) had a mid-ventricular pattern, and the remaining 2 patients (14.3%) had the coexistence of mid-ventricular and subepicardial LGEs. Furthermore, 3 patients (21.4%) presented atrial wall thickening and 13 patients (92.9%) were carriers of an atrial LGE. Three patients (21.4%) died during follow-up: one patient (7.1%) died due to a sudden cardiac death, while other two patients (14.3%) died due to non-cardiovascular causes.

### 3.2. 3D Software Analysis

The ADAS 3D software was applied in all the 14 patients in the final population. For every patient, the characterization of core zones and border zones was performed in each myocardial layer. In addition, the corridor module was applied in all patients in order to check for channels of slow conduction. When differentiating between core zones and border zones of scars, the latter was the most extensively represented in all patients. Additionally, in a large percentage of exams, the software identified the highest degree of core zone (*n* = 9; 64.3%) and border zone (*n* = 12, 85.7%) fibrosis among the epicardial layers (namely layers >50% of the myocardial thickness). Slow-conduction channels were found in 35.7% of patients (*n* = 5).

### 3.3. Outcomes

**Figure 5:**
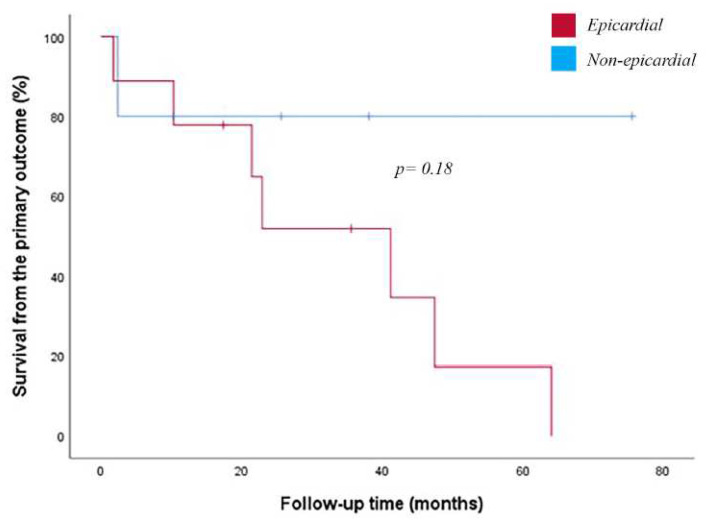
Survival analysis for the composite outcome. In red, patients with a higher degree of epicardial core zone fibrosis.

Among the baseline clinical characteristics taken into consideration, none were associated with adverse events. In addition, neither atrial LGE (*p* = 0.61) nor different ventricular LGE patterns were predictive of the primary outcome (isolated subendocardial LGE, *p* = 0.076; transmural LGE, *p* = 0.72; mid-ventricular LGE, *p* = 0.59; coexistence of mid-ventricular and subepicardial LGEs, *p* = 0.18). Notably, the presence of atrial wall thickening at baseline was predictive of the composite outcome according to the Kaplan–Meier analysis (*p* = 0.003) and, in this group, all patients experienced at least one event: one was hospitalized for acute heart failure, another patient underwent PMK implantation, and the last patient experienced an SCD. In addition, the Kaplan–Meier analysis found atrial wall thickening to be a predictor of the need for pacemaker implantation (*p* = 0.003) but not for the occurrence of VAs (*p* = 0.056). Despite their arrhythmogenic potential, the presence of corridors did not correlate with VAs (*p* = 0.46) nor with the primary outcome (*p* = 0.86). Interestingly, patients whose core zone fibrosis was mostly represented in epicardial layers (namely layers >50% of the wall thickness) had a tendency towards a higher incidence of the composite outcome, but without reaching statistical significance (*p* = 0.18; Figure 5) (Table 2).

## 4. Discussion

### 4.1. CMR Overview

Our study gives a current perspective of the CMR features in patients affected by all types of CA. This disease is one of the hot topics in modern cardiology and its prevalence has risen in recent years probably due to our improvement in diagnostic abilities. Different tools are applicable for such patients and the vast majority of diagnoses can be made through laboratory tests and bone scintigraphy. However, there may be difficulties when assessing a patient through such instruments as some amyloidotic forms are not detectable with bone scintigraphy and multiple patterns, as well as confounding factors, may hamper our identification of the disease. In all these circumstances, CMR imaging could come in handy; the current guidelines recommend its application in patients with negative test results and concomitant high clinical suspicion as well as in cases where a negative bone scintigraphy coexists with positive hematologic assays indicating the possibility of an AL form [1]. In this study, we tried to focus more on a different approach for CMR imaging. Our aim was to expand imaging indications not only in the diagnostic setting, but also to other cardiomyopathies and in the management and prognosis of patients affected by CA.

### 4.2. Ventricular LGE

As expected by the infiltrative mechanism of the disease, a huge number of patients presented ventricular LGEs and fibrosis. A subendocardial LGE was the most common pattern among our population; however, unlike other reports, the LGE localization was not predictive of adverse outcomes in our sample, probably due to its small size. Transmural LGEs were relatively rare (7.1% of patients) and this might have influenced their clinical relevance, thus justifying their low predictive value.

### 4.3. Atrial Amyloidosis

A particularly important point in this study is the atrial involvement of CA. Interestingly, the vast majority of patients had a detectable amount of atrial LGE, thus confirming the elevated prevalence of this parameter. An additional feature that has been less described in the literature is the presence of atrial wall thickening, which not only seems to be highly specific for CA, but it is also correlated with atrial dysfunction and thromboembolism, most likely related to the blood stagnation promoted by endothelial dysfunction and by the lack of atrial motility due to parietal infiltration and Bachmann bundle damage [14]. In our population, atrial LGE was common, but atrial wall thickening was less represented and, more importantly, it was significantly associated with the primary outcome. In addition, this parameter was predictive of the need for pacemaker implantation, but not of ventricular arrhythmias. This could be in line with the pathogenesis of such phenomena; we speculate that atrial thickening may be a marker of a deeper amyloid infiltration inside the atrial walls that might induce an earlier involvement of both the sino-atrial and atrio-ventricular nodes (due to anatomical proximity), consequently resulting in a conduction disease. Despite its statistical significance, the low number of patients prevents us from making wide inferences in the CA population and the role of this specific parameter still needs to be properly addressed.

### 4.4. Arrhythmia Prevention

Left ventricular EF still represents one of the major indicators for the implantation of a defibrillator (ICD) in many diseases. However, in patients affected by CA, an EF above 50% does not necessarily represent a predictor of a good prognosis. The presence of thickened walls combined with diastolic dysfunction grants these patients a good emptying performance of the ventricular cavity even in advanced phases of the disease. The current guidelines for the management of cardiomyopathies do not recommend the use of ICDs in primary prevention for patients affected by CA [1]. This statement is based on two studies showing that, in CA, ICDs are not beneficial for mortality and the presence of VAs is correlated with an increased death rate even in ICD carriers [15,16]. This means that VAs represent a marker of amyloid infiltration and their presence might correlate with an advanced stage of the disease. Thus, their occurrence is the alarm bell to search for morphological and structural abnormalities. For this reason, in our study, we also took into account the direct consequence of the amyloid burden, which is represented by scar tissue formation, which is detectable by the ADAS 3D software.

### 4.5. ADAS 3D

The ADAS 3D software carries the ability to reconstruct a three-dimensional map of the ventricular wall in its whole thickness by splitting it into different layers. For each layer, the software identifies and characterizes scar areas. In particular, the recognition of scar characteristics gives an indication regarding the extent, localization, and nature of the diseased tissue. Interestingly, the differentiation of fibrosis in core and border zones might be an additional feature that is able to provide further insights regarding arrhythmogenesis; we know that not only dense fibrotic areas (core zones), but also the “neighboring” ones, made up of both fibrotic and muscular tissues, can serve as a primary arrhythmogenic substrate by facilitating the creation of re-entry circuits [17,18]. In addition, the presence of corridors of slow conduction dispersed inside fibrotic areas may provide further information regarding the potential arrhythmogenic risk.

### 4.6. Ventricular Corridors and Scar Analysis

Border zones carry a heterogeneous structure that is able to organize itself into channels capable of conducting electrical stimuli; not by chance, the presence of channels inside fibrotic areas has already shown a strong association with sustained VAs [19] and the presence of fibrosis is one major mechanism responsible for arrhythmogenesis in patients with CA [20,21,22,23]. The presence of channels has already been identified as a useful tool currently under use for guiding ventricular tachycardia ablation [24], and recent data suggest that their reduction might represent a good indicator of a successful ablation procedure [25,26]. Sanchez-Somonte et al. found in a recent study that the presence of corridors detected by CMR was significantly associated with appropriate ICD interventions independently from the absolute value of EF and, conversely, a low scar burden and the absence of corridors have a high negative predictive value for ICD therapies [27]. However, these studies were not performed in CA patients and, therefore, our knowledge on this matter is still embryonic. By considering both the high incidence of VAs in patients affected by CA and the elevated scar burden in this population, we believe that the identification of these corridors might become a major element in the prediction of adverse outcomes even in patients affected by this cardiomyopathy. Unfortunately, in this case, their presence did not emerge as a valid predictor of VAs, but we must take into account that, in our small sample, VAs were a rare event (*n* = 1; 7.1%). Furthermore, it has already been demonstrated how, in CA, the amount of LGE correlates with a reduction in electrogram amplitudes in electro-anatomical voltage mapping and we know that the extension of dense scar areas confers a high prognostic burden for death and heart failure hospitalizations [28]. Our study is consistent with these observations; we found that patients with extensive core zone fibrosis (dense scar) in epicardial layers had a tendency towards a higher incidence of the primary outcome compared to those with a more extended pattern in subendocardial areas, but this association did not reach statistical significance. In this sense, apart from the aforementioned statistical limitations, we are also conscious that there may be discrepancies between imaging and electro-anatomical mapping when exclusively using LGE [29]. Our hope is that the application of ADAS 3D will overcome such limits while increasing our diagnostic and prognostic yield.

### 4.7. Study Limitations

There are some limitations in this study. The first one is the heterogeneity of the group, which included patients with ATTR-wt, ATTR-m, and AL amyloidosis, which we know are three different diseases with different pathogenesis processes and clinical presentations. In particular, patients affected by AL-CA present a higher incidence of VAs and their underrepresentation in the present cohort might have influenced the results. Another point is that the prognosis of AL-CA is often dictated by the presence of an underlying hematological disease and not only by the cardiomyopathy per se. Furthermore, the utility of CMR in the follow-up evaluation of CA is not well-established and its current applications cover a pure diagnostic role; thus, virtually all patients were in the very first phases of the disease at the time of the CMR imaging. This might have influenced the imaging results by showing a less severe involvement. Another point is that the application of novel therapies may influence the occurrence of adverse events by interfering with pathogenetic molecular mechanisms in a fashion that we are currently unable to predict. Moreover, this study was carried out at a single center with a high expertise in the management of cardiomyopathies and rare diseases; this might have affected the results by employing the most recent therapies and facilities, thereby reducing the incidence of adverse events, which were rare in our cohort. In addition, the retrospective design, the restricted follow-up time, and the low number of patients limits the possibility of large inferences in the CA population. However, despite its low statistical power, this study represents the first description of a possible application for the ADAS 3D software in the management and risk stratification of patients affected by CA and, from our perspective, by all types of cardiomyopathies.

## 5. Conclusions

CMR imaging is a fundamental instrument in the diagnosis and characterization of many cardiomyopathies. Its accuracy is like no other in the field of cardiac imaging and, in our opinion, it could be further enhanced by the employment of the ADAS 3D software. In CA, CMR is able of characterizing many different tissue properties and the possibility of scar analysis might improve our prognostic and, therefore, preventive abilities in a such unique disease. No definite conclusion can be made regarding CA from our study, but the evidence suggests that atrial wall thickening, ventricular scar evaluation, and corridors will have a growing role in determining the prognosis of both ischemic and non-ischemic cardiomyopathies. In this sense, this study represents, to our knowledge, the first application of the ADAS 3D software in CMR imaging of patients affected by CA.

## Figures and Tables

**Table 1:** Baseline characteristics of the final population. SD: standard deviation; EF: ejection fraction; MI: myocardial infarction; VTs: ventricular tachycardias; SVT: supraventricular tachycardia; PMK: pacemaker; ICD: implantable cardioverter defibrillator; ARBs: angiotensin receptor blockers; MRAs: mineralocorticoid receptor antagonists; SGLT-2: sodium–glucose cotransporter 2; ARNI: angiotensin receptor–neprilysin inhibitor.

Characteristic	Total (*n* = 14)
Age, years, mean ± SD	66.9 ± 14.8
Male gender, *n* (%)	11 (78.6)
CA variant, *n* (%)	
- ATTR-wt	8 (57.1)
- ATTR-m	1 (7.1)
- AL	5 (35.7)
Baseline EF, %, mean ± SD	54.7 ± 11.4
Anamnesis	
- Previous MI, *n* (%)	-
- Hypertension, *n* (%)	8 (57.1)
- Dyslipidemia, *n* (%)	7 (50.0)
- Diabetes, *n* (%)	3 (21.4)
- Smoking history	4 (28.6)
- Previous stroke, *n* (%)	-
- Previous VTs, *n* (%)	4 (28.6)
- Previous SVAs, *n* (%)	6 (42.9)
○ AF, *n* (%)	4 (28.6)
○ Typical AFL, *n* (%)	-
○ Atypical AFL, *n* (%)	2 (14.3)
- Previous PMK implantation	1 (7.1)
- Previous ICD implantation	1 (7.1)
Therapy at baseline assessment	
- Beta-blockers, *n* (%)	3 (21.4)
- ACE inhibitors, *n* (%)	2 (14.3)
- ARBs, *n* (%)	6 (42.9)
- MRAs, *n* (%)	2 (14.3)
- SGLT-2 inhibitors, *n* (%)	1 (7.1)
- Loop diuretics, *n* (%)	10 (71.4)
- ARNI, *n* (%)	-
- Tafamidis, *n* (%)	1 (7.1)
- Amiodarone, *n* (%)	4 (28.6)

**Table 2:** Imaging characteristics of the final population analyzed with the ADAS 3D software and their correlation with the primary outcome. LGE: late gadolinium enhancement.

Imaging Feature (*n*, %)	Correlation (*p*)
- Presence of atrial LGE (*n* = 13, 92.9%)	*p* = 0.61
- Mid-ventricular LGE (*n* = 1, 7.1%)	*p* = 0.59
- Mid-ventricular + subepicardial LGEs (*n* = 2, 14.3%)	*p* = 0.18
- Transmural ventricular LGE (*n* = 1; 7.1%)	*p* = 0.72
- Isolated subendocardial ventricular LGE	*p* = 0.076
- Atrial wall thickening (*n* = 3; 21.4%)	*p* = 0.003
- Presence of corridors (*n* = 5; 35.7%)	*p* = 0.86

## Data Availability

Restrictions apply to the datasets: The datasets presented in this article are not readily available because part of an ongoing study.
